# Supplementary material for: Patterns of Intron Gain and Loss in Fungi
Source: PLoS Biol. 2004 Nov 30;2(12):e422. doi: 10.1371/journal.pbio.0020422 (PMC532390; doi:10.1371/journal.pbio.0020422)
Supplement: Table S1 — Also available at http://genes.mit.edu/NielsenEtAl/. (4.3 MB ZIP). [file pbio.0020422.st001.zip › NielsenEtAl/html/1270.html]

AN0349.1.NCU05427.1.MG03187.1.FG04314.1


```
 CLUSTAL W (1.82) Multiple Sequence Alignments - Introns Inserted


Sequence 1: NCU05427.1	563 aa
Sequence 2: FG04314.1	604 aa
Sequence 3: MG03187.1	518 aa
Sequence 4: AN0349.1	630 aa
Alignment Length: 668 aa
Number Identitical Residues: 168 aa
Alignment Score (without introns) 9473


MG03187.1 	MSVFTRSCSSCPRLLTNHVATHAG-----------------SRSFSVSCRAPAASRRTQ-
NCU05427.1	--MFAARTPAS-----QHLAR--------------------LRYISYSSRLALSPRRSQ-
FG04314.1 	MAPFVRAYPTARCSL-CHVSRSIIRPVPPLTLRVSIHNTSLQRQHRYISTSQPSLRRNN-
AN0349.1  	MLRLPCRPLSVHRTALRYRPLAIHP-------------SHLRRGFSSSSVSFPTFTQFDR
          	   :     :       : .                  :   *     .    :  : : 

MG03187.1 	-DFG-SGYTSSYDP-EESGRGPMFS-KPSFGVPQFYPRDLKRRLDEFVVGQERAKKTTTS
NCU05427.1	-SYNNQGFSSSYDPNQENGRGPIFSNKASFGVPQFYPRDLKRRVDDYVVGQDRAKKTICS
FG04314.1 	-DFN-SGFSSSYDPSGDSGRGPMFS-KTNFGVPQFYPRDLKKRVDDYVVGQDRAKKTICA
AN0349.1  	SDFTSQPFSGVYET-GLPTAGPLGS-TPAFGV-RITPKSLKQYLDQFVVGQERAKKILSV
          	:.: .. ::. *:.      **: * .. *** :: *:.**: :*::****:****    

MG03187.1 	VVFNHYQ---GQRRRQNIEDE---RRVEEERLLRQQHFQR--------------------
NCU05427.1	VIFNHYQ---NIRRRQHHEIQ---DQRQREKLQRQRYARDHRDLQDRE------------
FG04314.1 	TIFNHYQ---NLRRRHQHDHE---DRNRRDKVMRQRFARDRELHQKRREMHPVEGQRAHS
AN0349.1  	AVYNHYQRVQELQRRQEEAEQLLAKRLRREDIQRRQEEREELLGKHAS------------
          	.::****  .  :**:.   :  : : ..: : *::  :     .               

MG03187.1 	----------------~----ARREPHP~--------------AEGQGHPE-SKLER1--
NCU05427.1	----------------~GYSGSSRDIHP0TPSVDD-----FYIPEDTNAPQRVKIDK~SN
FG04314.1 	SFSERYAADAIEDEFP~GHSESVRGLHD~SHEFDDDPMDHLYVTEDITVPEHVKIDK~SN
AN0349.1  	-----TDSVEHHPVED1EYPGQQRTIYP~NNPPTQP--SYATDNAEIDESSQLQIEK~SN
          	       :           .   *  :  .    .   .          ..  :::: :.

MG03187.1 	-----PTGVGKTYIVE2HL----NVPFTISDCNSFTQAGYIGQDVEACIERLLIEADYDV
NCU05427.1	LLLIGPTGVGKTYILE2TLSKKLNVPFTISDCNSFTQAGYIGQDVEACIERLLIEANYDV
FG04314.1 	LLLIGPTGVGKTYILE2TLSKKINVPFSICDCNSFTQAGYIGQDVETCIERLLIEANYDI
AN0349.1  	VLLLGPSGVGKTLMCR~SLARVLSVPFSISDCTPFTQAGYIGDDAEVCVHRLLAAANYDV
          	    .*:***** : .  *:   .***:*.**..********:*.*.*:.***  *:**:

MG03187.1 	NAAENGIVVLDEFDKLARRESVNGRDVGGEGVQQALLKLVEGSKVTVNVKDTRSSRSPSP
NCU05427.1	KAAEHGIVVLDEFDKIARRETVNGRDVGGEGVQQALLKLVEGTKVTVSVKDHRSSRPPPP
FG04314.1 	KATEYGIVVLDEFDKIARRETTTGRDVGGEGVQQALLKLVEGTKVTINVKDNRSSRSTPP
AN0349.1  	EQAERGIIVLDEIDKIAAAKVSHGRDVGGSGVQESLLKLLEGTTVQVQAKQERSAPRLSG
          	: :* **:****:**:*  :   ******.***::****:**:.* :..*: **:   . 

MG03187.1 	ITSNYNGG----SQTSQSSPPPGPRVEQ----ITIDTTNILFIFCGAFVGLDKIVLRRVA
NCU05427.1	PNLNISTPGYGPTSTTPSATP--GKVEQ----YIVDTSNILFVFCGAFVGLDKTVLRRVA
FG04314.1 	ITTNYNAS--GSSSSTPQANPPGGKVDQ----YTIDTKNILFVFCGAFVGLDKAVLRRIA
AN0349.1  	TTSSSYPPN-GPLGNTPFTPPGGGNVPHKGEVYNVRTDNILFICSGAFAGLHKVVMDRIS
          	 . .    . ..  .:  : * . .* :...   : * ****: .***.**.* *: *::

MG03187.1 	KPTIGFGSEPIRN----------GDALPREMYAHLPHQPH----------MVKPGTSG--
NCU05427.1	KPSIGFGAEVRNHR--GSSMSSSQDILPPELYSHLPHQPPTMPVDLSGGSLASHGAGG--
FG04314.1 	RPTMGFGGELRGR----SSMSGNKQTLPAETYTHLPHHNP-------------QSASS--
AN0349.1  	RGSMGFGQPVRNPPQTSTDRPHSYDSTANQPVPILPGSEEEALYKKHLPFFTPPSLSSPE
          	: ::***       .:.:. . . :  . :  . **        .         . ....

MG03187.1 	-----FTPLDLTTPADLQAFGFIPELIGRIHNICALTPLSLDELLRILTEPRNSLVAQYK
NCU05427.1	-----FTPLDLASPADLQAFGFIPELIGRLHNICALSPLSLDELYRILTEPRNSLVAQYT
FG04314.1 	-----FTPLDLTTPADLQSFGFIPELIGRLHNICALSPLSKEDLFRILTEPRNSLVAQYT
AN0349.1  	AEPTYFNALDLINQTDLQNYGFIPELVGRIPVNAALSALSQPLLVRILTEPRNSLVAQYT
          	:..: *..*** . :*** :******:**:   .**:.**   * **************.

MG03187.1 	ALFETYPSRLFFTSRALYAIAERAAKNETGARGLKMEMERVLAEPMFDAP---MPYVVID
NCU05427.1	ALFETYPSKLYFTRKALYAIAERAAKNETGARGLKMEMERVLAEPMYDAP---MPYVLIT
FG04314.1 	ALFETYPSRLFFTEKSLYAIAERAAASETGARGLKMEMERVLAEPMFDAP---MPYVLIT
AN0349.1  	TLFSLSGIELRFTTPALHKIAANAFTMGTGARALRTEMETILSDAMFETPGSSVKFVLVT
          	:**.    .* **  :*: ** .*    ****.*: *** :*::.*:::*.::: :*:: 

MG03187.1 	EACVKGTSKARYWGKDGRLDVDRLLREES-------------DMGQQT-----GDNASHS
NCU05427.1	EGCVKGTEKAGYWGKDGRLELERMMAEE--------------DQKGLG-----QGPVEGG
FG04314.1 	EACVKGTDKAGYWGKDGRFELDRRMQAE--------------EMHPAK-----ADP-DST
AN0349.1  	ENVAERKEKPVYLGRGQGSRFHSMIAAEEDRWEERMRQKKLKDRKGKSPLTFEDGPSISS
          	*  .: ..*. * *:.    ..  :  *..  ..   ... .:     . : . .     

MG03187.1 	YQPYREASQGGA
NCU05427.1	FERMREAGQSGA
FG04314.1 	FEGYRQAGQSGG
AN0349.1  	FKEYRSKASV--
          	::  *. ..
```
